# *Perilla* Seed Oil Enhances Cognitive Function and Mental Health in Healthy Elderly Japanese Individuals by Enhancing the Biological Antioxidant Potential

**DOI:** 10.3390/foods10051130

**Published:** 2021-05-19

**Authors:** Michio Hashimoto, Kentaro Matsuzaki, Shahdat Hossain, Tomoko Ito, Harumi Wakatsuki, Yoko Tanabe, Miho Ohno, Setsushi Kato, Kazuya Yamashita, Osamu Shido

**Affiliations:** 1 Department of Environmental Physiology, Faculty of Medicine, Shimane University, Izumo, Shimane 693-8501, Japan; matuzaki@med.shimane-u.ac.jp (K.M.); shahdat@juniv.edu (S.H.); harumiwa@med.shimane-u.ac.jp (H.W.); tanabey@med.shimane-u.ac.jp (Y.T.); o-shido@med.shimane-u.ac.jp (O.S.); 2 Department of Biochemistry and Molecular Biology, Jahangirnagar University, Savar, Dhaka 1342, Bangladesh; 3 Faculty of Nursing and Nutrition, The University of Shimane, Izumo, Shimane 693-8501, Japan; t-ito@med.shimane-u.ac.jp (T.I.); k-yamashita@u-shimane.ac.jp (K.Y.); 4 Department of Community Health and Gerontological Nursing, Faculty of Medicine, Shimane University, Izumo, Shimane 693-8501, Japan; 5 Kato Hospital, Jinjukai Healthcare Corporation, Kawamoto, Shimane 696-0001, Japan; ohno@k-jinju.or.jp (M.O.); katosetsu@k-jinju.or.jp (S.K.)

**Keywords:** *perilla* seed oil, α-linolenic acid, antioxidant potential, cognitive function, mental health, elderly people

## Abstract

Oxidative stress plays an important role in age-associated cognitive decline. We recently reported that dietary intake of *perilla* seed oil (PO), a rich source of α-linolenic acid (LNA, C18:3, ω-3), helps in maintaining good mental health in adults. This study aimed to investigate the impacts of dietary PO intake on cognitive functions and mental health in healthy, elderly Japanese individuals. Seventy-five healthy volunteers aged 64–84 years were randomly divided into two groups: a control group and a PO-administered group. At baseline and at 12 months of intervention, cognitive function, mental health condition, fatty acid profile of the red blood cell plasma membranes (RBC-PM), and serum biochemical parameters were evaluated. Results showed that serum biological antioxidant potential and LNA levels in the RBC-PM at 12 months after the trial were significantly higher in the PO group compared to the control group. Further, both the cognitive function measures, as evaluated by the Frontal Assessment Battery test and the apathy scores, tended to be improved after 12 months in the PO group. Our results demonstrate that dietary PO intake enhances the antioxidant potential and prevents the age-related cognitive and mental decline in healthy elderly individuals by enhancing the blood LNA levels.

## 1. Introduction

Aging is an inescapable process characterized by progressive deterioration of physiological integrity leading to functional impairment. Aging is related with cognitive impairment, and age-related memory and cognitive decline are associated with decreased plasma antioxidant levels and increased brain oxidative stress [1]. Although aging is a primary risk factor for several psychiatric disorders, epidemiological evidence suggests that, in humans, diet plays a major role in learning ability and mental health. For example, low levels of ω-3 polyunsaturated fatty acids (PUFA) in the plasma and the brain are associated with the development of dementia in the elderly and in patients with Alzheimer’s disease (AD) [2]. Further, epidemiological studies have shown that ω-3 PUFA deficiency impairs learning and memory [2,3], and is associated with age-related neurodegenerative disorders [3]. Although no noticeable effect of ω-3 PUFA supplementation has been observed in patients with AD, several reports suggest that chromic dietary ω-3 PUFA supplementation is effectual for some, but not all, elderly people with very mild cognitive impairment or with age-related cognitive decline [4,5,6]. Studies have shown that ω-3 PUFA-rich foods prevent age-associated cognitive decline in elderly Japanese individuals with cognitive deficits [7]. Therefore, we hypothesized that long-term supplementation of ω-3 PUFA such as α-linolenic acid (LNA), could prevent some of the harmful effects of aging on brain function in the healthy elderly.

Compared to other edible vegetable oils, *perilla* seed oil (PO) has the highest proportion (54–64%) of LNA (C18:3), which is an essential ω-3 PUFA [8]. LNA is converted to other ω-3 PUFAs such as eicosapentaenoic acid (EPA, C20:5) and docosahexaenoic acid (DHA, C22:6) in the mammalian liver. However, in humans, the metabolic conversion of LNA to other ω-3 PUFAs, particularly DHA, is very limited [9]. Therefore, it is important to investigate whether dietary LNA could supply EPA and DHA to various tissues. It has been reported that LNA is an antithrombotic [10], antiarrhythmic [11], anti-inflammatory [12], and neuroprotective [13] fatty acid. PO also possesses antidepressant activity, and PO intake improves cognitive ability in rats by producing new hippocampal neural membrane structures as well as by inducing the expression of specific proteins involved in several functions, including regulation of the cytoskeleton, energy metabolism, transport, neurogenesis, and apoptosis [14]. We recently reported the potential palliative impacts of PO on psychological conditions such as apathy and depression in healthy Japanese adults [15]. These studies suggest that LNA has protective effects against age-associated cognitive and mental deterioration in the older adults. However, detailed reports of the impacts of PO on cognitive ability and mental condition in the elderly are lacking. Therefore, this study aimed to investigate the impacts of PO rich in LNA on cognitive function and mental health in elderly Japanese individuals.

## 2. Materials and Methods

### 2.1. Subjects

This 12-month, single-blinded, randomized, parallel-armed study was conducted among 64–84-year-old healthy elderly individuals living in Shimane Prefecture, Japan. All participants underwent a medical examination including anthropometry, a blood biochemical assessment, and cognitive-function and mental-health tests. Participants having respiratory, hepatic, renal, or cardiac disease; those having diabetes mellitus; those with endocrine, metabolic, or hematological diseases; those using any psychotropic drug/supplement that might significantly affect the results of the study; and those who had an allergy or hypersensitivity were excluded.

This study was performed following the principles of the Declaration of Helsinki and Good Clinical Practice, and the protocol was approved by the Shimane University Ethics Committee (Study No.: 1889, 2128). Written informed consent was obtained from all participants prior to participation. This interventional study was conducted during the period between 2015 and 2017. Participants were inquired to answer a self-reported general lifestyle questionnaire, which included questions related to their medical/medication history.

The participants were assigned to two groups: the control group (n = 33) and the PO group (n = 42). Participants in the control group received 7.0 mL of canola oil (The Nisshin OilliO Group, Ltd., Tokyo, Japan) daily, and those in the PO group received 7.0 mL of PO (O-san farm Co., Kawamoto, Shimane, Japan) daily for 12 months. The fatty acid profiles of the canola oil and PO are shown in Table 1.

### 2.2. Anthropometry, Body Composition, and Dietary Intake Analysis

Body weight, height, and abdominal circumference of all participants were measured. Body composition was evaluated by a bioelectrical impedance analyzer WB-150 (TANITA Co., Tokyo, Japan). To evaluate the effects of PO intake on health status and lifestyle during the trial, the participants were asked to respond to a general questionnaire that included questions on medical/medication history and their lifestyle. 

Dietary intake before and after the interventional study was estimated by a validated, brief-type self-administered diet history questionnaire (BDHQ) for the Japanese elderly [16]. The BDHQ is a four-page questionnaire with fixed portion sizes that inquiries about the consumption frequency of selected foods to estimate the dietary intake of 58 food and beverage items in the previous month. The food and beverage items registered on the BDHQ were foods frequently consumed in Japan, mainly selected from a food list used in the National Health and Nutrition Survey of Japan. Based on the participant’s response to the BDHQ, the ad hoc computer algorithm assessed the amount of 98 nutritional factors intake during the previous month.

### 2.3. Blood Sampling 

At baseline and after 3, 6, and 12 months of the trial, blood samples were collected either in the morning or afternoon, after ascertaining that the participants had not eaten breakfast or lunch, respectively. Then, blood samples were separated into serum and erythrocyte (red blood cell (RBC)) aliquots. RBC samples were stored at −80 °C within 8 h of collection until use. RBC samples were collected to monitor the fatty acid profile of the RBC plasma membranes (RBC-PM). Fresh serum samples were used to measure blood biochemistry and biological antioxidant potential (BAP) levels. The remaining serum samples were stored at −80 °C within 8 h of collection to measure serum brain-derived neurotropic factor (BDNF) levels.

### 2.4. Evaluation of Cognitive Functions and Mental Health

Cognitive functions were evaluated by the Hasegawa’s Dementia Scale-Revised (HDS-R) [17], Mini-Mental State Examination (MMSE) [18], and Frontal Assessment Battery (FAB) [19]. Apathy was measured using the Japanese version of the apathy scale [20]. Depression was assessed using the Zung Self-Rating Depression Scale (SDS) [21].

### 2.5. Blood Biochemical Analysis, Fatty Acid Profile, and Apolipoprotein E Genotyping

Biochemical analyses included evaluation of the levels of gamma-glutamyl transpeptidase, alanine aminotransferase, aspartate aminotransferase, albumin, total cholesterol, blood urea nitrogen, triglycerides, creatinine, blood sugar, and high- and low-density lipoprotein cholesterol using an automatic analyzer BiOLis 24i (Tokyo Boeki Medical System, Tokyo, Japan). In addition, hemoglobin A1c was determined by a commercially available kit (TFB Inc., Tokyo, Japan). 

Serum BDNF level was quantified using a BDNF Emax ImmunoAssay System kit (Promega Inc., Madison, WI, USA) according to the company’s protocol. 

Serum BAP level was determined using a free radical elective evaluator (FREE Carpe Diem) that included a spectrophotometric device reader, and measurement kits (BAP test; Wismerll Co. Ltd., Tokyo, Japan) were optimized to the FREE Carpe Diem System according to the manufacturer’s protocol. 

The fatty acid profile in the RBC-PM was measured by direct transmethylation as previously described [5,22].

Apolipoprotein E (*APOE*) genotyping was performed as previously described [7]. APOE single nucleotide polymorphism genotyping was performed by a real-time polymerase chain reaction (7900HT; Applied Biosystems, Waltham, MA, USA) using the TaqMan single nucleotide polymorphism genotyping assay for rs429358 and rs7412 with identification numbers G_3084793-20 and C_904973-10, respectively (Applied Biosystems Waltham, MA, USA).

### 2.6. Statistical Analysis

The Shapiro–Wilk test was carried out to evaluate the distribution of the data. Data are expressed as the mean ± SE. Changes in FAB (∆FAB) and apathy (∆Apathy) scores and participants’ parameters at baseline and after 12 months of intervention were analyzed using the Mann–Whitney U test. LNA, EPA, and DHA levels in the RBC-PM, fatty acid profile of the RBC-PM, and serum BAP and BDNF levels were analyzed using repeated measures analysis of variance (ANOVA) followed by Bonferroni’s post hoc test. The correlations between FAB scores and BAP, and between ∆BAP and ∆Apathy scores were evaluated using the Pearson’s correlation coefficient. All analyses were performed using the PASW Statistics software (version 23.0, SPSS Inc., Chicago, IL, USA). All statistical tests were two-tailed, and significance was set at *p* < 0.05.

## 3. Results

### 3.1. Demographic and Clinical Characteristics of Participants and Assessment of Dietary Intake

Seventy-five participants (40 women and 35 men) were randomly allocated to either the control group or the PO group. Baseline characteristics are shown in Table 2. Serum albumin levels were significantly greater in the PO group compared to the control group. Blood sugar and HbA1c levels were significantly lower in the PO group in comparison to the control group. The remaining parameters showed no significant differences between the two groups (Table 2).

The frequency of different APOE alleles was measured in both the groups. The frequency of the APOE2/2 or APOE2/3 genotype was 21 (70.0%) in the control group and 31 (77.5%) in the PO group. The frequency of the APOE2/4 or APOE3/4 genotype was 8 (26.7%) in the control group and 7 (17.5%) in the PO group. The frequency of the APOE4/4 genotype was 1 (3.3%) in the control group and 2 (5.0%) in the PO group. These results indicate that there were no significant differences in the distribution of different APOE alleles between the two groups. 

**Figure 1:**
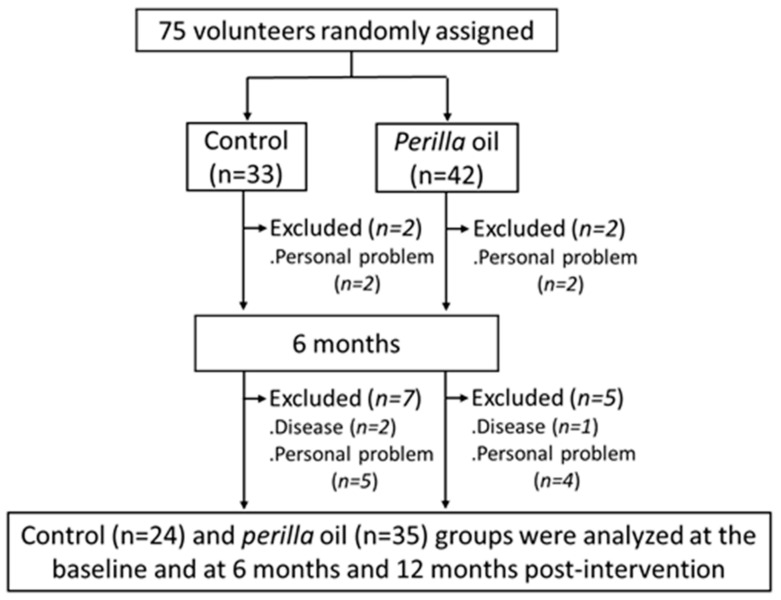
Flow diagram of participant selection.

The 12-month study was completed by 24 control group subjects and 35 PO group subjects (Figure 1). Data acquired from the general questionnaire on lifestyle habits and medical/medication history at baseline and after 12 months of intervention showed no differences. There were no noticeable side effects (allergic reaction, palpitations, stomach irritation, etc.) that affected the daily lives of the participants that were noted in either group.

No significant differences between the two groups in dietary intake based on responses to the BDHQ at either baseline or after 12 months were found (Table 3), indicating that PO intake for 12 months did not affect the dietary intake and nutritional values.

Participant characteristics after 12 months of intervention are shown in Table 4. Serum HbA1c levels were significantly lower in the PO group compared to the control group. The remaining parameters showed no significant differences between the two groups (Table 4). The mean changes (∆) in the anthropometry, blood pressure, and blood biochemical parameters including serum albumin, blood sugar, and HbA1c after 12 months of intervention were not significantly different between the two groups (data not shown), indicating that PO intake did not influence the renal function, hepatic function, or lipid metabolism.

### 3.2. Cognitive Functions and Mental Health Assessment

**Figure 2:**
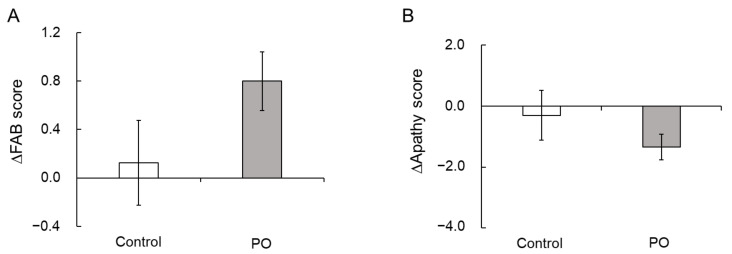
Effect of PO-intake on FAB and apathy scores. Mean changes (Δ) in the FAB scores (**A**) and apathy scores (**B**) after 12 months of intervention in the PO and control groups. Bar represents mean ± SE. PO, *perilla* seed oil; FAB, Frontal Assessment Battery.

The mean HDS-R, MMSE, and FAB scores of all participants (n = 75) at baseline were 27.9 ± 0.3/30, 28.3 ± 0.2/30, and 15.6 ± 0.2/18, respectively (Table 2). At baseline and after 12 months of intervention, after adjustment for serum albumin, blood sugar, and HbA1c, the total scores were not different between the two groups (Table 2 and Table 4). However, the mean change (∆) in the FAB scores from baseline to month 12 was only slightly higher (*p* = 0.094) in the PO group than in the control group (Figure 2A). 

At baseline and after 12 months, there were no significant differences in the apathy and SDS scores between the two study groups (Table 2 and Table 4). However, the mean change (∆) in the apathy scores from baseline to month 12 tended to be lower in the PO group compared to the control group (*p* = 0.100, Figure 2B).

### 3.3. Fatty Acid Profile of the RBC-PM

The RBC-PM fatty acid profiles at baseline and after 12 months are indicated in Table 5. LNA levels markedly increased over time in the PO group; the other RBC-PM fatty acid levels did not alter. The RBC-PM fatty acid profile remained unchanged over time in the control group. This increase in the mean LNA levels of the RBC-PM further supported the consumption compliance in the PO group subjects.

**Figure 3:**
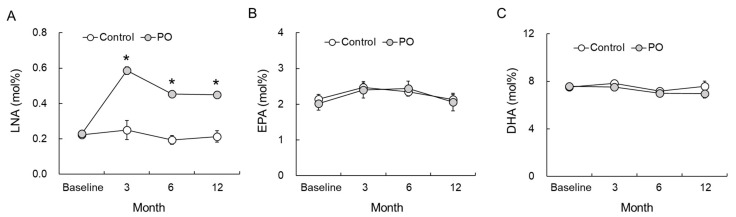
Effect of dietary PO on fatty acid levels in the RBC-PM of the PO-intake and control groups. (**A**) LNA, (**B**) EPA, and (**C**) DHA levels at 3, 6, and 12 months after intervention. Control group, n = 24–33; PO group, n = 35–42. Results are mean ± SE. * *p* < 0.05. LNA, linolenic acid; EPA, eicosapentaenoic acid; DHA, docosahexaenoic acid; PO, *perilla* seed oil-intake group; RBC-PM, red blood cell plasma membranes.

LNA, EPA, and DHA levels in the RBC-PM assessed at baseline and at 3, 6, and 12 months after the treatment are shown in Figure 3. LNA levels increased significantly at 3, 6, and 12 months in the PO group (Figure 3A). Further, compared to the control group, LNA levels were significantly higher in the PO group at all time points (F = 11.307, *p* < 0.0001). EPA (Figure 3B, F = 0.152, *p* = 0.929) and DHA (Figure 3C, F = 0.067, *p* = 0.523) levels in the RBC-PM remained unchanged during the intervention and showed no significant difference between the two groups at all time points.

### 3.4. Biological Antioxidant Potential (BAP) and Brain Derived Neurotropic Factor (BDNF) Levels

**Figure 4:**
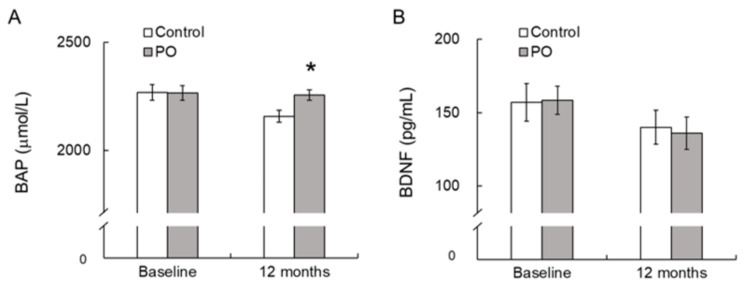
Effect of dietary PO on serum BAP (**A**) and BDNF levels (**B**) in the PO-intake and control groups. Values are mean ± SE. * *p* < 0.05, compared with the control value. Control group, n = 24–33; PO group, n = 35–42. BAP, biological antioxidant potential; BDNF, brain-derived neurotrophic factor; PO, *perilla* seed oil-intake group.

At baseline, there were no significant differences in serum BAP and BDNF levels between the two groups. At 12 months, serum BAP levels were significantly greater in the PO group compared to the control group (Figure 4A). Serum BDNF levels showed no difference between the two groups at baseline and at 12 months after the trial (Figure 4B).

**Figure 5:**
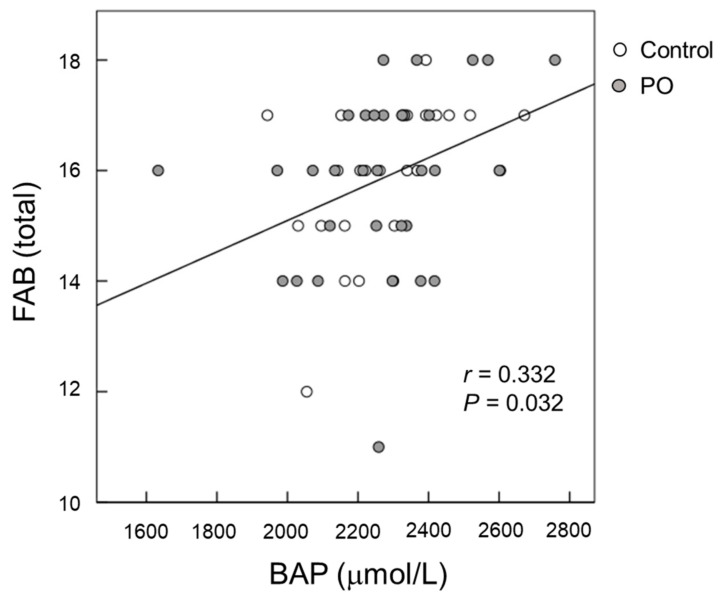
Scatter plot of the relationship between FAB scores and serum BAP at baseline. (Open circle) control group; (Grey circle) *perilla* seed oil-intake group. FAB, Frontal Assessment Battery; BAP, biological antioxidant potential.

**Figure 6:**
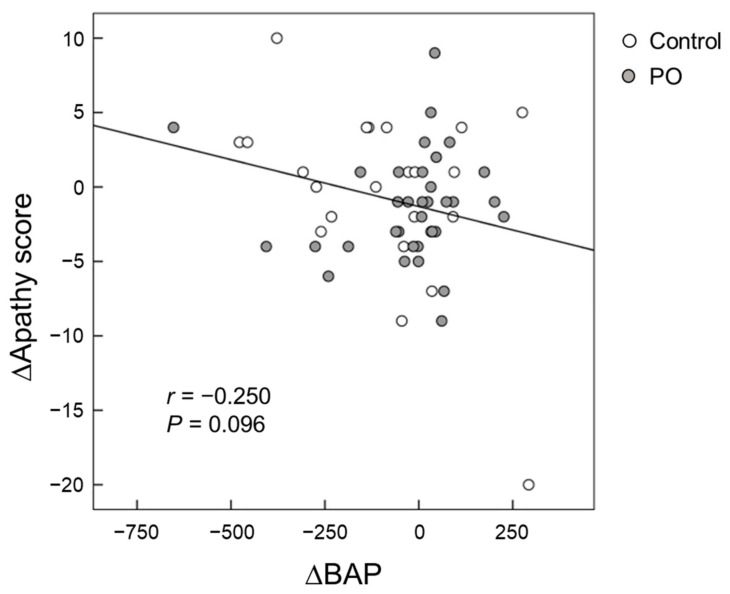
Scatter plot of the relationship between changes in apathy scores and changes in serum BAP from baseline to month 12. (Open circle) control group; (Grey circle) *perilla* seed oil-intake group. BAP, biological antioxidant potential.

Serum BAP levels were positively correlated (*p* = 0.032) with FAB scores at baseline after adjustment for serum albumin, blood sugar, and HbA1c (Figure 5). For all participants, the change (∆) in the serum BAP from baseline to month 12 tended to be negatively correlated (*p* = 0.096) with the change (∆) in apathy scores from baseline to month 12 after adjustment for serum albumin, blood sugar, and HbA1c (Figure 6).

## 4. Discussion

Our results suggest that daily dietary PO supplementation increases blood LNA levels and BAP and helps in maintaining optimal cognitive function and good mental health in Japanese elderly individuals with age-related cognitive decline. Further, our study also suggests that dietary PO intake is not associated with any clinically significant side effects. It has been shown that the natural ingredients of the PO are nontoxic [23]. To the best of our knowledge, this is the first long-term (12 months), randomized, controlled trial to evaluate the influence of PO intake on cognitive functions and mental health in 64–84-year-old healthy Japanese individuals with age-related cognitive decline. All the participants were in excellent compliance during the study period, as the RBC-PM LNA levels were significantly greater in the PO group compared to the control group at 12 months (Table 2).

LNA, an essential PUFA, possesses pharmacological activities, including anti-cardiovascular, antioxidant, and neuroprotective activities [24,25,26]. In our study, the change in the mean FAB score after 12 months of intervention was only slightly higher in the PO group than in the control group (*p* = 0.094) (Figure 2A), suggesting a possible protective effects of the PO diet against age-related cognitive decline in the elderly. Contrary to our results, Kamalashiran et al. [27] reported that the use of PO along with standard neuroprotective therapy in patients with mild to moderate dementia does not improve cognitive function. The discrepancy between our results and those reported by Kamalashiran et al. might be because of differences in the study participants and PO dose. Kamalashiran et al. targeted patients with an MMSE score of 10–23, and the PO dose was 3 g/day, while we targeted healthy elderly individuals with an MMSE score of 25 or higher, and the PO dose used in our study was 7 g/day.

The brain is sensitive to oxidative balance, and oxidative stress is involved in the initiation and progression of cognitive impairment. It has been shown that cognitive impairment is significantly associated with oxidative stress, and enhancing the antioxidant system may protect cognitive ability in the elderly [1]. We observed that after 12 months of intervention, serum BAP levels were significantly greater in the PO group compared to those in the control group (Figure 4). Serum BAP, which reflects the total antioxidant capacity, is a generally used biomarker of oxidative stress and can be easily measured in large populations [28]. In our study, serum BAP levels were positively correlated with the FAB score at baseline (Figure 5), suggesting that dietary antioxidants may protect against age-related cognitive decline in the elderly. This observation corresponds with a previous report by Pesce et al. [29] that showed that improvements in memory and global cognitive function were significantly related with increased resistance to oxidative stress at the plasma level in healthy elderly people. Vitamin C, vitamin E, and polyphenols are well-known antioxidants [30]. PO contains vitamin E (Table 1) and other antioxidants [31], which might have contributed to the increased serum BAP in the PO-administered participants. Taken together, the positive effects of PO intake on age-associated cognitive decline in healthy elderly individuals may be related to its antioxidant activity, causing increased resistance against oxidative stress.

Lee et al. [14] showed that, in rats, ameliorated spatial learning and memory upon supplementation of a LNA-rich *perilla* diet is corelated with the differential expression of hippocampal proteins. Functional annotation revealed that these differentially expressed proteins are involved in several important functions, including regulation of the cytoskeleton, energy metabolism, transport, neurogenesis, and apoptosis. This suggests that dietary LNA supplementation induces changes in the membrane fatty acid composition, leading to improved cognitive function. Moreover, the beneficial effects of LNA on learning and memory in an Aβ25-35-infused AD murine model are comparable to those exerted by DHA [32]. In rodents, ingestion of ω-3 PUFA including LNA increases the DHA and EPA levels in the hippocampus, leading to beneficial effects on brain functions [33]. PO mediates neuroprotection via its antioxidative actions against dementia, as PO is rich in LNA [34]. However, the metabolic conversion of LNA to ω-3 PUFA, particularly DHA, in humans is very limited [9,35]. Normally, an increase in the levels of dietary components in the RBC-PM reflects their incorporation into the cell membranes of other tissues. Therefore, their increased levels could be contributing to several physiological activities, including brain function. In the present study, LNA intake did not change the DHA and EPA levels in the RBC-PM of the elderly (Figure 3B,C). Therefore, it is not clear from our results whether the protective action of a PO diet against age-related cognitive decline in the elderly is the effect of LNA alone or the combined effect of LNA with DHA and/or EPA. LNA can be easily oxidized and is a preferred substrate for ketogenesis [36]. Freemantle et al. [37] suggested that LNA could induce mild ketonemia aimed at retaining or restoring cognitive function in the elderly, and that the use of LNA as a mildly ketogenic fatty acid might improve neuronal function. Altogether, these findings suggest that the protective action of the PO diet against age-correlated cognitive decline in the elderly is a direct effect of LNA. However, further studies are required to support our observation.

Apathy scores are a measure of motivational engagement [19], while SDS scores reflect the degree of depression [20]. The symptoms of motivational engagement and depression usually overlap. The relevance between the brain lipids and mental function remains unclear. In the present study, the mean changes in apathy scores after 12 months of intervention tended to be larger in the PO group than that of the control group (*p* = 0.100) (Figure 2B), suggesting an improvement in apathy. This result is consistent with that of our recent study in healthy Japanese adults that showed the potential palliative effects of PO on mental conditions such as depression and apathy [15]. Apathy reduces the quality of life in the elderly and in patients with neurological disorders such as dementia and AD. Su et al. [38] has shown that ω-3 fatty acids have mood-regulating and antidepressant effects in humans. We previously reported that long-term supplementation of dietary ω-3 PUFA-enriched meals improves apathy in elderly Japanese individuals with cognitive impairment [7]. In this study, serum BAP was found to be negatively correlated with apathy scores (*p* = 0.032, Figure 5), suggesting that antioxidants supplementation could reduce apathy in the elderly. Taken together, our results showed that PO intake improves apathy in both adults and the elderly, presumably by reducing oxidative stress.

Although studies showed an association between serum BDNF levels and cognitive functions [39,40], results have been inconsistent. Hakansson et al. [41] reported that the capability to respond to physical exercise with increased serum BDNF concentration is correlated with cognitive function, and the ability to produce peripheral BDNF is a marker of BDNF availability in the brain. Moreover, administration of ω-3 PUFA increases serum BDNF levels in Aβ-infused AD model rats [42] and during the first episode of schizophrenia [43]. The relationship between ω-3 PUFA intake and increased serum BDNF may contribute to the effectiveness of ω-3 PUFA on cognitive function. These results suggest that dietary PO administration may influence serum BDNF levels in elderly individuals with age-related cognitive decline. In our study, however, PO intake had no effect on serum BDNF levels in healthy elderly individuals (Figure 4).

In the present study, compared to baseline, the LNA levels in the RBC-PM after 3, 6, and 12 months of trial were significantly increased in the PO group (Figure 3A). By contrast, the levels of EPA and DHA in the RBC-PM of the PO group did not alter during the trial (Figure 3B,C). Our results are consistent with those of Hamazaki et al. [44], who reported that PO intake does not influence EPA and DHA levels of the human plasma phospholipid fraction. Contrary to these results, we recently reported that in Japanese subjects, PO intake significantly increases both LNA and EPA levels in the RBC-PM after the intervention, but the DHA levels remain unaffected [15]. In both of our interventional studies, the amount of PO administered was the same (7.0 mL/day PO, which is equivalent to about 4 g LNA/day) for both adults and the elderly, yet the results were different. Studies showed that LNA is not converted to DHA in all cases [9,35], and there is a difference between adults and the elderly with respect to their abilities of converting LNA to DHA. The initial conversion of LNA to 18:4 ω-3 by the action of ∆6-desaturase is the rate-limiting reaction of the pathway in which LNA is metabolically converted to EPA and DHA. Burdge and Wootton [45] indicated that the conversion of LNA to EPA in women aged approximately 28 years was markedly greater (2.5-fold) than that in men of a similar age, suggesting a sex-associated difference in the activity of the desaturation-elongation pathway. Thus, the pathway might be regulated by sex hormones, particularly estrogen [46]. In the present study, however, this pathway appears to be less affected by sex hormones since the women participants were 64–84 years old, and thus, sex differences seem to be irrelevant. Studies showed that in mice, the levels of ∆6-desaturase in the liver decrease with age (40% decrease between 4 and 17 months of age) [47], suggesting that an age factor might be involved in LNA metabolism. Therefore, differences in the RBC-PM EPA levels might be due to the age difference of the subjects, although the clear mechanisms are unknown. Taken together, our results showed that LNA improves cognitive function and mental condition in the elderly without significantly increasing the brain EPA and DHA levels. Further studies are needed to understand the underlying molecular mechanism.

The *APOE-ε4* allele has been consistently shown to be the strongest and most prevalent genetic risk factor for sporadic late-onset AD, and individuals carrying the *APOE-ε4* allele have an increased risk of developing AD [48,49]. Because of the pleiotropic nature of *APOE* possession, the *APOE-ε4* allele can have deleterious effects by influencing multiple biological processes, including lipid metabolism, amyloid beta deposition, neuro-inflammation, neurogenesis, and synaptic function [50,51]. This suggests that the penetrance of the *APOE-ε4* allele influences the rate of cognitive dysfunction and the likelihood of transitioning to mild cognitive impairment (MCI) and AD, which is variable and potentially modifiable [52,53]. Therefore, since the difference in the *APOE-ε4* allele frequency may affect the results of the present interventional study, it is meaningful to measure the allele frequency. In this study, there were no significant differences in the distribution of different *APOE-ε4* alleles between the control and PO groups. Thus, it seems that the difference in frequency of *APOE-ε4* alleles of participants did not affect the outcome in this interventional study.

In the present study, serum albumin, blood sugar, and HbA1c levels at baseline were significantly different between the control and PO groups (Table 2). Since participants in this study were randomly assigned into two groups at the baseline, not all outcomes may be the same between the two groups. We are unsure whether these differing outcomes would affect the value of this study. Several epidemiological studies have reported that serum albumin [54] and HbA1c [55] are associated with cognitive decline in the elderly. Thus, these were analyzed as confounding factors when comparing the impacts of PO intake on cognitive ability between the control and PO groups. Additionally, we evaluated the changes (∆) in these outcomes from baseline to month 12.

Our study has several limitations. First, since the subjects in this study were healthy elderly people without cognitive impairment and mental illness, the decline in their cognition and mental health parameters were mostly age-related, and, hence, there were no significant differences between the PO and control groups. Future studies are required to examine the impacts of dietary PO intake on subjects with cognitive impairment and mental illness including dementia and depression. Second, the sample size in our study was small with participants from a localized population, which limits the generalizability of our findings. Third, a total of 16 subjects (9 in the placebo group, 7 in the PO group) dropped out during the 12-month intervention period due to illness or personal reasons (Figure 1). As dropouts may have a significant impact on the composition of the sample and in turn the results, care should be taken in interpreting the results. Fourth, although the group assignment and the type of oil distributed were not disclosed to the subjects, they may have been able to distinguish the type of oil by taste and odor. Moreover, placebo effects may be included in behavioral measurements in this single-blind study. In the future, it may be necessary to conduct a double-blind study in a state where the taste and odor of oil cannot be distinguished, such as by using encapsulated oil. Fifth, we had no information on the genetic background of the participants and did not continue follow-up after the termination of the study.

## 5. Conclusions

Dietary PO intake increases serum LNA levels and BAP in healthy elderly Japanese individuals. Moreover, PO intake mitigates the age-related cognitive and mental decline in elderly individuals without any harmful side effects. These effects might be the result of increased antioxidant potential due to PO intake. Further studies are required to elucidate the mechanisms underlying the beneficial effects of PO on cognitive function and mental health.

## Figures and Tables

**Table 1:** Basic fatty acid component of canola oil and *perilla* seed oil.

Fatty Acids (g/100 g)	Canola Oil	*Perilla* Seed Oil
Palmitic acid (C16:0)	4.3	5.6
Stearic acid (C18:0)	2.09	1.7
Oleic acid (C16:0, ω-9)	61.7	12
Linoleic acid (C18:2, ω-6)	19	13.9
α-linolenic acid (C18:3, ω-3)	9.14	62.2

*Perilla* seed oil (PO) also contains vitamin E (67.8 mg/100 g). Data on canola oil were obtained from the United States Department of Agriculture (USDA) (https://fdc.nal.usda.gov/fdc-app.html#/food-details/1103863/nutrients, (accessed on 14 January, 2017)), and data on PO were obtained from Japan Food Research Laboratories (JFRL).

**Table 2:** Participants’ baseline parameters.

	Control (n = 33)				PO (n = 42)				
	Mean	Median	Min	Max	Mean	Median	Min	Max	*p* Value
*Anthropometry*									
Sex (male/female)	16/17				19/23				
Age (years)	71.0	71.3	64	84	71.0	70.5	65	83	0.988
Height (cm)	155.3	153.8	137.0	173.3	155.9	155.3	136.0	175.0	0.776
Body Weight (kg)	55.5	58.3	34.6	75.1	54.9	53.5	36.5	80.8	0.818
BMI (kg/m^2^)	22.9	22.4	15.4	28.6	22.4	22.3	16.9	32.5	0.641
BC (cm)	84.4	84.6	64.0	100.0	82.5	82.0	63.0	116.0	0.409
Body fat (%)	26.5	26.2	10.9	38.5	26.5	25.3	16.8	36.3	0.993
*Blood pressure*									
SBP (mmHg)	148.1	147.0	116.0	198.0	148.1	147.0	106.0	198.0	0.995
DBP (mmHg)	81.3	81.0	57.0	102.0	84.1	83.0	49.0	107.0	0.312
*Cognitive index*									
HDS-R score	28.1	29.0	22.0	30.0	27.8	28.0	18.0	30.0	0.503
MMSE score	28.4	29.0	24.0	30.0	28.3	29.0	22.0	30.0	0.412
FAB score	15.7	16.0	11.0	18.0	15.5	16.0	8.0	18.0	0.507
*Emotional index*									
SDS score	32.6	31.0	20.0	49.0	32.9	31.5	22.0	52.0	0.869
Apathy score	10.3	11.0	1.0	24.0	10.3	10.0	2.0	23.0	0.981
*Blood biochemistry*									
GOT (U/L)	25.6	25.0	17.0	43.0	26.1	26.0	16.0	40.0	0.695
GPT (U/L)	22.6	19.0	8.0	64.0	21.0	19.0	12.0	39.0	0.465
γ-GT (IU/L)	39.7	22.0	11.0	365.0	36.6	23.0	8.0	167.0	0.787
ALB (g/dL)	4.3	4.3	3.4	4.9	4.4	4.4	4.1	5.1	0.002
TC (mg/dL)	208.0	207.0	161.0	296.0	215.8	215.0	144.0	294.0	0.305
TG (mg/dL)	112.7	98.0	53.0	255.0	118.6	108.0	50.0	308.0	0.636
BUN (mg/dL)	16.0	15.0	9.5	26.1	15.9	15.2	10.4	35.7	0.867
CRE (mg/dL)	0.7	0.7	0.5	1.5	0.7	0.7	0.5	1.6	0.824
BS (mg/dL)	113.8	99.0	84.0	252.0	97.2	94.0	69.0	191.0	0.018
HDL-C (mg/dL)	60.5	59.0	32.0	99.0	65.7	62.0	40.0	113.0	0.202
LDL-C (mg/dL)	124.9	112.0	68.0	210.0	126.2	129.0	73.0	184.0	0.838
HbA1c (NGSP) (%)	6.2	5.9	5.2	10.7	5.7	5.6	5.2	6.8	0.004

ALB: albumin; BC: belly circumference; BMI: body mass index; BUN: blood urea nitrogen; BS: blood sugar; CRE: creatinine; DBP: diastolic blood pressure; FAB: Frontal Assessment Battery score; GPT: glutamate-pyruvate transaminase; GOT: glutamate-oxaloacetate transaminase; γ-GT: gamma-glutamyl transpeptidase; HbA1c: glycated hemoglobin; HDL-C: high-density lipoprotein cholesterol; HDS-R: Hasegawa’s dementia scale-revised; LDL-C: low density lipoprotein cholesterol; MMSE: Mini-Mental State Examination; NGSP, National glycohemoglobin standardization program; n: number; PO: *perilla* seed oil-intake group; SBP: systolic blood pressure; SDS: self-rating depression scale; TC: total cholesterol; TG: triglyceride; Min: minimum value; Max: maximum value. *p*-value: control vs. PO.

**Table 3:** Dietary intake evaluated by the BDHQ before and after intervention in the control and *perilla* seed oil-intake groups.

		Baseline		Month 12		Month 12—Baseline		
	Unit	Control (n = 33)	PO (n = 42)	Control (n = 24)	PO (n = 35)	Control (n = 24)	PO (n = 35)	*p*-Value
Energy	KJ/d	8774 ± 565	9302 ± 493	8326 ± 454	8560 ± 1905	−448 ± 473	−742 ± 371	0.670
Protein	g/d	84.4 ± 4.8	92.0 ± 6.4	79.6 ± 5.6	83.6 ± 5.3	−4.7 ± 5.9	−8.4 ± 3.5	0.452
Fat	g/d	63.9 ± 4.3	65.2 ± 4.6	58.5 ± 4.2	58.1 ± 3.9	−5.4 ± 3.7	−7.1 ± 2.8	0.704
Carbohydrate	g/d	277.3 ± 22.5	279.9 ± 15.4	266.9 ± 15.9	262.9 ± 14.8	−10.4 ± 18.5	−17.0 ± 14.7	0.779
Total dietary fiber	g/d	15.0 ± 1.1	15.6 ± 1.0	14.8 ± 1.3	15.7 ± 1.1	−0.2 ± 1.3	0.1 ± 0.9	0.842
Saturated fat	g/d	22.3 ± 1.3	17.8 ± 1.3	15.3 ± 1.2	15.3 ± 1.1	−2.0 ± 1.0	−2.5 ± 0.8	0.713
Monounsaturated fat	g/d	15.4 ± 1.6	22.8 ± 1.7	20.7 ± 1.6	20.5 ± 1.5	−1.6 ± 1.4	−2.3 ± 1.1	0.668
Polyunsaturated fat	g/d	15.0 ± 0.9	15.4 ± 1.1	14.7 ± 1.0	13.9 ± 0.9	−0.7 ± 0.9	−1.4 ± 0.7	0.524
ω-6 polyunsaturated fat	g/d	11.8 ± 0.7	11.6 ± 0.8	11.5 ± 0.8	10.6 ± 0.7	−0.4 ± 0.6	−1.0 ± 0.5	0.507
ω-3 polyunsaturated fat	g/d	3.5 ± 0.3	3.7 ± 0.3	3.2 ± 0.2	3.3 ± 0.2	−0.3 ± 0.3	−0.5 ± 0.2	0.667
LNA (C18:3ω-3)	g/d	1.84 ± 0.12	1.82 ± 0.13	1.84 ± 0.14	1.69 ± 0.12	0.005 ± 0.1	−0.13 ± 0.09	0.353
EPA (C20:5ω-3)	g/d	0.51 ± 0.06	0.59 ± 0.06	0.41 ± 0.04	0.48 ± 0.04	−0.098 ± 0.06	−0.11 ± 0.05	0.919
DHA (C22:6ω-3)	g/d	0.84 ± 0.09	0.96 ± 0.10	0.68 ± 0.06	0.74 ± 0.06	−0.16 ± 0.10	−0.16 ± 0.07	0.976

Values are means ± SE. BDHQ: brief-type self-administered diet history questionnaire; d, day; DHA: docosahexaenoic acid; EPA: eicosapentaenoic acid; LNA α-Linolenic acid; PO: *perilla* seed oil-intake group. *p*-value: control vs. PO.

**Table 4:** Participants’ parameters after 12 months intervention.

	Control (n = 24)				PO (n = 35)				
	Mean	Median	Min	Max	Mean	Median	Min	Max	*p*-Value
*Anthropometry*									
Sex (male/female)	12/12				16/19				
Age (years)	72.0	71.3	65	85	72.0	70.8	66	84	0.883
Height (cm)	154.6	154.7	136.0	170.5	156.3	155.7	136.4	175.0	0.502
Body Weight (kg)	55.9	59.9	38.1	77.9	54.4	52.5	35.2	81.0	0.512
BMI (kg/m^2^)	23.3	23.1	17.5	29.8	22.1	21.7	16.3	32.1	0.100
BC (cm)	85.7	86.5	65.5	103.0	82.0	82.6	63.0	115.0	0.103
Body fat (%)	26.1	25.4	6.7	41.4	25.4	24.4	14.6	42.2	0.578
*Blood pressure*									
SBP (mmHg)	143.6	136.0	104.0	188.0	135.7	139.0	99.0	174.0	0.396
DBP (mmHg)	79.3	76.7	56.0	106.0	76.5	76.3	55.0	99.0	0.552
*Cognitive index*									
HDS-R score	28.7	28.8	26.0	30.0	29.1	29.3	26.0	30.0	0.194
MMSE score	29.0	29.0	25.0	30.0	29.2	29.4	26.0	30.0	0.865
FAB score	16.0	16.2	12.0	18.0	16.5	16.8	13.0	18.0	0.223
*Emotional index*									
SDS score	32.3	30.3	21.0	54.0	31.9	31.0	22.0	51.0	0.547
Apathy score	9.5	9.0	0.0	19.0	8.4	8.1	0.0	26.0	0.383
*Blood biochemistry*									
GOT (U/L)	24.7	24.3	18.0	46.0	26.1	24.0	16.0	60.0	0.792
GPT (U/L)	19.2	16.3	11.0	46.0	20.1	17.7	10.0	53.0	0.501
γ-GT (IU/L)	31.6	19.3	13.0	172.0	29.6	23.5	11.0	102.0	0.677
ALB (g/dL)	4.2	4.2	3.4	4.6	4.3	4.3	3.9	4.9	0.134
TC (mg/dL)	196.0	196.0	124.0	255.0	205.1	202.0	144.0	279.0	0.342
TG (mg/dL)	105.1	91.0	51.0	264.0	108.1	82.0	41.0	402.0	0.605
BUN (mg/dL)	16.5	15.7	11.3	24.5	16.6	16.0	9.8	23.6	0.896
CRE (mg/dL)	0.8	0.7	0.5	1.9	0.8	0.7	0.5	1.5	0.694
BS (mg/dL)	105.7	98.0	88.0	166.0	100.9	97.3	73.0	216.0	0.290
HDL-C (mg/dL)	57.4	56.3	31.0	80.0	64.9	64.3	40.0	94.0	0.068
LDL-C (mg/dL)	117.9	112.5	70.0	167.0	119.3	124.0	49.0	191.0	0.796
HbA1c (NGSP) (%)	6.2	6.0	5.3	7.9	5.8	5.7	5.2	6.9	0.020

ALB: albumin; BC: belly circumference; BMI: body mass index; BUN: blood urea nitrogen; BS: blood sugar; CRE: creatinine; DBP: diastolic blood pressure; FAB: Frontal Assessment Battery score; GPT: glutamate-pyruvate transaminase; GOT: glutamate-oxaloacetate transaminase; γ-GT: gamma-glutamyl transpeptidase; HbA1c: glycated hemoglobin; HDL-C: high-density lipoprotein cholesterol; HDS-R: Hasegawa’s dementia scale-revised; LDL-C: low density lipoprotein cholesterol; Max: maximum value; Min: minimum value; MMSE: Mini-Mental State Examination; NGSP: National glycohemoglobin standardization program; n: number; PO: *perilla* seed oil-intake group; SBP: systolic blood pressure; SDS: self-rating depression scale; TC: total cholesterol; TG: triglyceride; *p*-value: control vs. PO.

**Table 5:** Fatty acid profile of the red-blood cell plasma membranes at baseline and after 12 months of intervention.

	Baseline		12-Months		
	Control (n = 33)	PO (n = 42)	Control (n = 24)	PO (n = 35)	*p*-Value
*Fatty acid (mol%)*					
PLA	25.8 ± 0.2	25.6 ± 0.2	23.6 ± 0.5	24.0 ± 0.4	0.557
POA	0.4 ± 0.0	0.4 ± 0.0	0.5 ± 0.1	0.5 ± 0.0	0.591
STA	17.8 ± 0.2	17.7 ± 0.2	17.5 ± 0.3	18.0 ± 0.3	0.284
OLA	14.7 ± 0.5	14.6 ± 0.5	15.8 ± 0.3	15.9 ± 0.3	0.430
LLA	11.3 ± 0.3	11.6 ± 0.3	11.7 ± 0.3	11.7 ± 0.5	0.965
LNA	0.2 ± 0.0	0.2 ± 0.0	0.2 ± 0.0	0.4 ± 0.0	<0.0001
AA	10.5 ± 0.3	11.0 ± 0.3	10.8 ± 0.5	10.3 ± 0.5	0.513
EPA	2.1 ± 0.2	2.0 ± 0.1	2.1 ± 0.2	2.1 ± 0.1	0.774
DPA	2.0 ± 0.1	2.0 ± 0.0	1.5 ± 0.1	1.5 ± 0.1	0.681
C24:0	3.7 ± 0.1	3.6 ± 0.1	4.7 ± 0.1	4.8 ± 0.1	0.702
DHA	7.5 ± 0.2	7.6 ± 0.2	7.6 ± 0.4	7.0 ± 0.4	0.295
C24:1	3.8 ± 0.1	3.7 ± 0.1	3.9 ± 0.1	3.8 ± 0.1	0.587

Values are mean ± SE. AA: arachidonic acid; DHA: docosahexaenoic acid; DPA: docosapentaenoic acid; EPA: eicosapentaenoic acid; LLA: linoleic acid; LNA: α-linolenic acid; n: number; OLA: oleic acid; PLA: palmitic acid; PO: *perilla* seed oil-intake group; STA: stearic acid. *p*-value is control vs. PO.

## Data Availability

The data presented in this study are available upon reasonable request from the corresponding author.
